# Morbid risk of schizophrenia amongst relatives of schizophrenia probands: A family-controlled study

**DOI:** 10.4102/sajpsychiatry.v24i0.1173

**Published:** 2018-11-26

**Authors:** Justus U. Onu, Jude U. Ohaeri, Ngozi N. Unaogu, Chikaodili M. Inechi, Nwamaka B. Nweze, Vincent N. Ubochi, Jojo U. Onwukwe

**Affiliations:** 1 Department of Clinical Services, Federal Neuropsychiatric Hospital, Nigeria; 2 Department of Psychological Medicine, University of Nigeria, Nigeria

## Abstract

**Introduction:**

There is a dearth of data on heritability of schizophrenia in Africa. The few African studies that addressed familial psychiatric morbidity in schizophrenia involved relatively small sample sizes and addressed psychiatric morbidity only in first-degree relatives. The present study sought to improve upon the methodology of previous African studies, and widen the scope to second- and third-degree relatives with a view to enriching the field of genetic epidemiology in Africa.

**Methods:**

This study elicited information on the morbid risk of schizophrenia amongst 5259 relatives of schizophrenia probands (*n* = 138) and 6734 relatives of healthy controls (*n* = 138) through direct interview of patients, available relatives of patients and controls. Diagnosis of probands was confirmed using Mini International Neuropsychiatric Interview. Through a direct interview of 138 patients and their available relatives, a family history approach using the Family Interview for Genetic Studies was utilised to obtain information on the morbid risk for all relatives that could be recalled. The same approach was utilised for the interview of the controls (aged 45 years and above) and their relatives. Morbid risk estimates were calculated using the Weinberg shorter method.

**Results:**

Morbid risk for schizophrenia in the first-, second- and third-degree relatives of schizophrenia probands was 10.9% (95% confidence interval [CI] = 10.6–11.2), 4.2% (95% CI = 4.1–4.3) and 3.9% (95% CI = 3.6–4.2), respectively, compared with 2.6% (95% CI = 2.5–2.7), 1.6% (95% CI = 1.5–1.7) and 1.5% (95% CI = 1.4–1.6), respectively, of the healthy control group.

**Conclusion:**

The findings support the widely noted impression that schizophrenia significantly aggregates in families of schizophrenia probands more than healthy controls.

## Introduction

In genetic research, it is of more interest to know the lifetime prevalence than the point prevalence; but even lifetime prevalence is not a wholly satisfactory statistic because some family members at the time of study will be unaffected but may develop the disorder at a future date, and others may have already died unaffected.^1^ The concept of lifetime morbid risk has been used to address these limitations. Assessment of lifetime morbid risk is an integral part of the epidemiologic assessment of many diseases and disorders.^2^ Lifetime morbid risk is usually defined and determined by the Kaplan-Meier product limit estimator^3^ and Weinberg method^4,5^ as the probability of a person developing a disorder during a specified period of his or her life or up to a specified age.

Schizophrenia is a heterogeneous group of disorders affecting about 1% of the general population.^6,7^ The disorder is largely considered to have a genetic basis, with genetic factors and gene-environment interactions contributing over 80% of the liability to schizophrenia.^8,9^

Data concerning family history of psychiatric disorders are often used to assist diagnosis, to examine the role of genetic or non-genetic familial factors in aetiology, to develop new methods of classification^10^ and to identify at-risk family members for possible prevention and early intervention, with a view to shortening the duration of untreated disease.^11^ Family studies have also been instrumental in establishing diagnostic validity and familial aggregation of schizophrenia.^12^ Information concerning familial prevalence may be collected by two different methods: The family history method (obtaining information from the patient or a relative concerning all family members) and the family study method (interviewing directly as many relatives as possible concerning their own present or past symptomatology). Other important methodological considerations in family studies include the different diagnostic procedures (best estimate diagnosis or not) and the different instruments of phenotype assessment (structured or not).

The indication is that 19.1% of first-degree relatives (FDRs) of patients with first-episode psychosis have been found to have psychosis.^13^ With respect to specific diagnoses, Chang et al.^14^ reported a morbid risk for schizophrenia amongst FDRs of 2.5% (Weinberg shorter method) or 3.9% (Kaplan-Meier estimate) – both figures being significantly higher than the 0.8% found in the general population. Kendler reviewed well-designed family studies of schizophrenia and he found that schizophrenia strongly aggregated in families with a relative risk of about 11.0% as compared with matched control groups, and there is no evidence that such familial aggregation differs across samples.^15^

There is paucity of data on heritability of schizophrenia in Africa. It is worthwhile to note that majority of previous studies were conducted in Western populations. In Africa, only a few studies addressed familial psychiatric morbidity in schizophrenia.^16,17,18^ Gureje et al.^17^ reported a morbid risk for schizophrenia of 4.12% in the relatives of probands with schizophrenia compared with 1.42% in the relatives of probands with mania. This is similar to the findings of Adamson and Okewole.^16,18^ These studies involved relatively small sample sizes (36–50 subjects) and addressed psychiatric morbidity only in the FDRs. The present study improved upon the methodology of previous African studies and added to the literature in the following ways: Firstly, we used a larger sample size than previous African studies and examined morbid risk in the first-, second- and third-degree relatives – a rare occurrence in the literature. Secondly, several reliable family members were interviewed to obtain the family history for each patient. Thirdly, the control group consisted of subjects who were beyond the known risk age for schizophrenia (≥ 45 years), thereby ensuring that the morbid risk obtained from this control group was more representative of those who were least likely to develop the disorder later in life, a situation which could affect the validity of comparing the two groups.^1^

## Materials and methods

### Study design and population

This was a controlled family history study with a cross-sectional design. The study was carried out amongst the in-patients of the Federal Neuropsychiatric Hospital, Enugu, Nigeria. The control group consisted of healthy staff of all cadres at the Enugu North Local Government Secretariat. Enugu North Local Government is one of the local councils located within Enugu metropolitan city in Enugu State, Nigeria.

Consecutive in-patients with the diagnosis of schizophrenia, who gave permission for their relatives to be approached, were recruited with their relatives into the study. Patients aged 18–64 years whose diagnosis was made at least 1 year prior to sampling (to allow for stability of diagnosis) were included in the study. Patients with schizophrenia of suspected organic aetiology or with medical or psychiatric co-morbidities were excluded through a detailed medical history, mental state examination using the diagnostic instrument Mini International Neuropsychiatric Interview (MINI) and full physical examination (including neurological examination). Patients with substance-use disorder were excluded, using the screening section of the MINI. We did not perform toxicological tests.

Only in-patients were involved because of the lengthy time of interview and also the fact that the admission period was an opportune time to locate many family members, especially during visit hours. Patients were interviewed when they were in stable clinical condition, that is, fully conscious, could follow the interview process and not requiring emergency chemical and/or physical restraint. Once clinical stability was established, adequate information about the nature of the study and the voluntary nature of their participation was provided to the patients, whilst the fact that they could withdraw from the study at any stage without any compromise to their care was reiterated. They demonstrated good comprehension of the study and made their independent decision to participate.

The comparison group consisted of healthy local government workers (not including health staff) aged above 45 years. This was to ensure that the comparison group had passed the maximal risk period and were less likely to develop the disorder under investigation (the maximal risk period for schizophrenia is 15–45).^1^

### Sample selection

#### Patients

Study subjects from the various wards were recruited as listed in the hospital’s admission register, which is a record of patients by date of admission and provisional diagnosis. A total of 250 patients were admitted into the various wards in the 6-month period of the study which was from 01 October 2015 to 31 March 2016. One hundred and sixty had a clinical diagnosis of schizophrenia by the unit consultant. They were approached and after complete description of the study, five declined to give consent because of unwillingness to participate. The remaining 155 were recruited for the study after obtaining written informed consent. They were reassessed and 138 met the criteria for schizophrenia using the *International Classification of Diseases and Related Health Problems*, 10th edition (ICD-10). Fifteen of the 155 subjects were excluded because of the presence of comorbid medical illnesses and/or substance-use disorders. An additional two were excluded because the illness had not lasted for up to 1 year.

In computing the required sample size for the study, we used the findings of Maier et al.^19^ for the following reasons: Firstly, they studied first-, second- and third-degree relatives and healthy control group. Secondly, they used similar instruments to obtain family history information. Thirdly, our comparison group was also from the community. Based on their finding that the morbid risk of schizophrenia in FDRs was 5.0%, compared with 0.8% for the control group, we computed the required sample size using Cochran formula for comparison groups (2*z*^2^*pq*/*d*^2^) and arrived at 124, each, for patients and comparison group.^20^

#### The control group

Workers with a major psychiatric diagnosis or history of chronic medical illnesses, such as diabetes mellitus, epilepsy, hypertension and duodenal ulcer were excluded. This was done using the screening sections of the MINI to screen for the presence of mental disorders and thorough medical history to exclude the presence of physical conditions.

## Procedure and measurement

### Diagnostic interview

Diagnosis of schizophrenia was made using the ICD-10 criteria for schizophrenia and confirmed using the MINI. One senior resident doctor (JUO) in psychiatry applied the MINI. He was trained to apply the study instruments by a senior psychiatrist with experience in the use of these instruments. At the preliminary stage of the study, the research team took turns in interviewing patients not involved in the study; and the study commenced when the senior research psychiatrist judged that the interviewers could confidently apply the questionnaires. Joint rating sessions were done periodically throughout the study to ensure that the standard interview process was being adhered to.

The family interview for patients, patients’ relatives and the control group was done by another set of two senior psychiatry residents (NBN and CMI), similarly trained by the senior psychiatrist, using the Family Interview for Genetic Studies (FIGS). They used the constructed family tree as a guide, to review the family pedigree of the participants, and then used the FIGS to collect family information. Patients’ relatives who looked after the patients in the ward or who intermittently visited the patients were, in a convenient room within the ward, interviewed directly using the Diagnostic Interview for Genetic Studies (DIGS). The information on relatives who could not be reached by the research interviewers was obtained through proxy interview (i.e. obtaining the history of the unavailable relatives by interviewing available relatives) using the FIGS. For the comparison group, all the family information was gathered via proxy interview.

For every first-, second- and third-degree relative with symptoms suggestive of schizophrenia, the research interviewer made a detailed summary. Two consultant psychiatrists reviewed all available information (DIGS, FIGS, other relevant side information and copies of medical records) pertaining to the probands’ relatives and the relatives of the control group. Best estimate lifetime psychiatric diagnosis according to the ICD-10 criteria was determined independently and then finalised by consensus. Two levels of diagnoses were possible: None and definite.

### Data analysis

The statistical analysis was done using the Statistical Package for Social Sciences, version 18. Results were first calculated as frequencies. Group comparisons were done using chi-square test (categorical variables) and one-way analysis of variance, where appropriate (continuous variables). All tests were two-tailed, and the level of significance was set at *p* < 0.05. The Weinberg method for age correction was used to calculate the lifetime at risk (morbid risk) for each group of relatives.^21^ In this article, we present the data for morbid risk related to the diagnosis of schizophrenia only.

## Ethical consideration

Ethical approval was obtained from the Ethical Committee of the Federal Neuropsychiatric Hospital Enugu, Enugu state, Nigeria, with reference number FNHE/HCS&T/REA/VOL.1/176. International ethical norms and standards were strictly adhered to in this study. Written informed consent was obtained from all the participants (patients, relatives directly interviewed and the control group). The patients underwent an adequate process of being informed about the research, the voluntary nature of their participation, their right to refuse to participate, whilst their ability to comprehend, make a decision and indicate their decision was ensured. For the relatives whom we did not directly interview, we gathered only non-intrusive personal characteristics, anonymously, viz., estimated age, gender, relationship with the proband or control and presence of mental disorder, all of which are in line with the routine experience of history taking in medicine. By the protocol of the ethical approval for this study, the information is guaranteed to be used only within the premises of the stated objectives of the study and will not be shared with anyone outside of those premises.

## Results

A total of 276 subjects (138 schizophrenia patients, 138 healthy controls) and their 11 993 relatives (5259 of patients and 6734 for the control) participated in the study. The socio-demographic profile of the 276 subjects is presented in Table 1. As expected from the methodology, the healthy comparison group was significantly older (49.1 years, standard deviation [s.d.] ± 3.1) than the patient group (31.8 years, s.d. ± 10.4) (*p* < 0.001) and were more likely to be married (*p* < 0.001).

**TABLE 1:** Sample characteristics.

Demographic variables	Patients (*n* = 138)		Control (*n* = 138)		Statistical differences
	*n*	%	*n*	%	
**Age†**	-	-	-	-	*t* = −18.734; *df* = 274; *p* = 0.001
**Gender**					
Male	73	52.9	59	42.8	*χ*^2^ = 2.846; *df* = 1; *p* = 0.092
Female	65	47.1	79	57.2	
**Marital status**					
Never married	106	76.8	24	17.4	*χ*^2^ = 105.108; *df* = 2; *p* < 0.001
Married	21	15.2	102	73.9	
Separated	11	8.0	12	8.7	
**Religious affiliation**					
Christianity	136	98.6	132	95.7	*χ*^2^ = 2.060; *df* = 1; *p* = 0.151
Other religions	2	1.4	6	4.3	
**Level of education**					
No formal education	3	2.2	0	0.0	*χ*^2^ = 32.963; *df* = 3; *p* < 0.001
Primary education	18	13.0	12	8.7	
Secondary education	80	58.0	43	31.2	
Tertiary and above	37	26.8	83	60.1	
**Employment status**					
Unemployed	100	72.4	0	0.0	*χ*^2^ = 158.278; *df* = 2; *p* < 0.001
Working part-time	10	7.2	22	16.0	
Working full-time	28	20.4	116	84.0	

† , Patients means – 31.76 ± 10.40; Control means – 49.07 ± 3.11.

The socio-demographic characteristics of the first-, second- and third-degree relatives of the probands and the comparison group are shown in Table 2. There was no significant difference in the mean age of the first-, second- and third-degree relatives of the probands and the control. As Table 2 shows, the frequency distribution of kinship characteristics for both groups was similar. The number of FDRs of the patients who were less than 15 years and more than 45 years of age were 136 and 350, respectively; whilst those for the control group were, respectively, 160 and 374. The prevalence of schizophrenia in the first-, second- and third-degree relatives of the probands with schizophrenia and control are presented in Table 3. The comparative prevalence of schizophrenia amongst FDRs (i.e. probands vs. control group) was 6.7% versus 1.5%; amongst second-degree relatives (SDRs), it was 3.0% versus 1.1%; and amongst TDRs, it was 2.3% versus 1.0% (*p* < 0.001). From these figures and the rest of Table 4, using the method of calculation cited in the section on methodology, the morbid risk was calculated.

**TABLE 2:** Socio-demographic details of the first-, second- and third-degree relatives of patients and control.

Variables	Probands		Control		Statistical differences
	*n*	%	*n*	%	
**FDRs†**					
Mean age of FDRs (s.d.)	36.85	1.63	36.50	1.62	*t* = 0.11, *df* = 2269, *p* = 0.92
Number of parents	276	25.3	276	23.3	χ^2^ = 4.99, *df* = 3, *p* = 0.17
Number of siblings	690	63.2	736	62.2	
Number of children	125	11.5	168	14.2	
**SDRs‡**					
Mean age of SDRs (s.d.)	45.12	3.65	43.5	5.49	*t* = 0.68, *df* = 4946, *p* = 0.51
Number of uncles	571	25.8	574	21.1	χ^2^ = 40.14, *df* = 4, *p* < 0.001
Number of aunts	471	21.3	656	24.1	
Number of nephews	357	16.1	519	19.1	
Number of nieces	280	12.7	422	15.5	
Number of grandparents	548	24.8	550	20.2	
**TDRs§**					
Mean age of TDRs (s.d.)	36.1	6.23	41.3	7.59	*t* = 0.54, *df* = 4772, *p* = 0.62
Number of paternal first cousins	926	47.5	1408	49.6	χ^2^ = 1.83, *df* = 1, *p* = 0.18
Number of maternal first cousins	1015	52.1	1425	50.2	

FDRs, first-degree relatives; SDRs, second-degree relatives; TDRs, third-degree relatives; s.d., standard deviation.

† , Probands – *n* = 1091, Control – *n* = 1180;

‡ , Probands – *n* = 2227, Control – *n* = 2721;

§ , Probands – *n* = 1941, Control – *n* = 2833.

**TABLE 3:** Comparison of prevalence of schizophrenia between families of probands and controls, for first-degree relatives, second-degree relatives and third-degree relative.

Variable	FDRs		Statistics	SDRs		Statistics	TDRs		Statistics
	Probands	Control		Probands	Control		Probands	Control	
Cases	73	18	*χ*^2^ = 39.33 *df* = 1 *p* < 0.001	67	31	*χ*^2^ = 22.04 *df* = 1 *p* < 0.001	45	29	*χ*^2^ = 12.653 *df* = 1 *p* = 0.001
Non-cases	1018	1162		2160	2690		1896	2804	
**Total**	**1091**	**1180**		**2227**	**2721**		**1941**	**2833**	

FDRs, first-degree relatives; SDRs, second-degree relatives; TDRs, third-degree relatives.

**TABLE 4:** Morbid risk estimates for first-, second- and third-degree relatives of schizophrenia probands and healthy control group.

Variables	Probands	Control
**FDRs with schizophrenia**		
*n*	73	18
%	6.7	1.5
BZ (schizophrenia) of FDRs	670	705
**Morbid risk**		
%	10.9	2.6
SE	0.16	0.6
95% CI	10.6–11.2	2.5–2.7
Total FDRs of schizophrenia (*n*)	1091	-
Total FDRs of healthy (*n*)	-	1180
**SDRs with schizophrenia**		
*n*	67	31
%	3.0	1.1
BZ (schizophrenia) of SDRs	1584	1888
**Morbid risk**		
%	4.2	1.6
SE	0.07	0.05
95% CI	4.1–4.3	1.5–1.7
Total FDRs of schizophrenia (*n*)	2227	-
Total FDRs of healthy (*n*)	-	2721
**TDRs with schizophrenia**		
*n*	45	29
%	2.3	1.0
BZ (schizophrenia) of TDRs	1156.5	1953
**Morbid risk**		
%	3.9	1.5
SE	0.17	0.07
95% CI	3.6–4.2	1.4–1.6
Total FDRs of schizophrenia (*n*)	1941	-
Total FDRs of healthy (*n*)	-	2833

FDRs, first-degree relatives; SDRs, second-degree relatives; SE, standard error; TDRs, third-degree relatives; BZ, Bezugsziffer; CI, confidence interval.

The morbid risk estimates of schizophrenia for the relatives of the patients were 10.9% (95% confidence interval [CI] = 10.6–11.2), 4.2% (95% CI = 4.1–4.3) and 3.9% (95% CI = 3.6–4.2), respectively, compared with 2.6% (95% CI = 2.5–2.7), 1.6% (95% CI = 1.5–1.7) and 1.5% (95% CI = 1.4–1.6), respectively, for the relatives of the healthy control group. The differences between the respective comparison groups are statistically significant because the 95% CIs do not overlap (Table 4).

## Discussion

The general aim of the study was to assess the morbid risk of schizophrenia amongst close relatives of schizophrenia probands, in comparison with a healthy control population. The major contribution of this study to the literature is the use of a more rigorous methodology in comparison with previous studies from Africa^16,17,18^ in particular, and many other studies from abroad, in general.

The socio-demographic profile of the probands shows that they were mostly similar to those of subjects from other Nigerian studies^16,17,18^ The finding that schizophrenia patients were significantly more likely to be single (76.8%) than the comparison group (17.4%) is explained not only by the difference in age but also by the well-reported tendency of the patients to remain single because of difficulty in forming close social bonds.^22^

In line with this, the number of children of the schizophrenia patients was fewer (125 [11.5%]) compared to the healthy control group (168 [14.2%]). This supports the finding that schizophrenia patients may remain single and so have fewer children. An earlier study in Egypt by Mansour et al.^23^ found that individuals with schizophrenia may have significant impairment in fertility and fecundity, placing them at a reproductive disadvantage. This tendency of schizophrenia patients to remain single and have fewer children may equally account for the fewer FDRs of patients with schizophrenia who were below the age of risk for schizophrenia, compared with the control group (136 vs. 160). It may be argued that the differences in the socio-demographic profile of the probands and the control might have affected the morbid risk of schizophrenia in their relatives; this is not likely as the socio-demographic characteristics of the relatives were similar (Table 2).

### Morbid risk of schizophrenia

The morbid risk of schizophrenia in the first-, second- and third-degree relatives of schizophrenia probands were 10.9% (95% CI = 10.6–11.2), 4.2% (95% CI = 4.1–4.3) and 3.9% (95% CI = 3.6–4.2), respectively. These figures are significantly higher than those of the corresponding relatives of the healthy control group (2.6%, 1.6% and 1.5%, respectively), as evidenced by the fact that the respective 95% CIs did not overlap. This finding supports the robust evidence in the literature, which suggests that schizophrenia strongly aggregates in families of schizophrenia probands more than the general population.^18,24^ It also supports the views of previous authors that the risk in relatives is a function of the degree of genetic relatedness to the probands and the comparison group.^24,25^ Thus, the highest risk was amongst the FDRs followed by second- and third-degree relatives, respectively. Our findings in this regard are in line with the literature.^21,26^

The morbid risk of schizophrenia in the FDRs of schizophrenia probands in this study (10.9%) was high compared to other family studies done in Africa using similar methodology. Earlier studies in Africa by Okewole et al.^18^ Adamson^16^ and Gureje et al.^17^ reported morbid risk of 4.12% – 6.7% amongst the FDRs of schizophrenia patients. There are some possible explanations for the higher morbid risk of schizophrenia in the present study, compared with the previous African studies.

Firstly, the proportion of directly interviewed relatives of probands in this study was about 25%. Although this was smaller than that of the previous family studies in Western and Asian countries (85% and 57%, respectively),^12,19^ it is still higher than that of other African studies that identified only one family member to obtain the family history of their relatives. The present study interviewed all the relatives present and also sourced information from the medical records of any affected relatives that had contact with the hospital. Hence, the methodology of this study, including the instruments used, had greater potential to identify more affected family members than the previous African studies. Secondly, the sample size of this study was much larger than those of previous African studies. For example, the previous Nigerian studies interviewed 36 and 50 probands, respectively, compared with 138 for the current study.^17,18^

However, it is noteworthy that the 10.9% morbid risk from this study is much similar to those of many findings from abroad.^15,26^ In other words, the morbid risk of schizophrenia in this study is at the upper limit of what is seen in the literature. For example, Kendler et al. and Verma et al. found rates as high as 11.0% and 16.97%, respectively, amongst FDRs of schizophrenia probands.^21,26^ In these studies, the methodology was similar to the present study in the following ways: Firstly, the same instrument was used to collect family history information. Secondly, at least one key family informant was interviewed, and both first- and second-degree relatives were included.

However, despite the robust evidence in the literature suggesting that the morbid risk of schizophrenia is higher in relatives of schizophrenia probands than the general population, this finding has been challenged by some authors who failed to show elevated risks in FDRs of schizophrenia probands.^27,28^ It is noteworthy that these studies with contrary findings have a number of methodological shortcomings which cast some doubt on the validity of the findings. In particular, small numbers of subjects and unsatisfactory diagnostic methods may have resulted in low power to detect differences between relatives and control populations.^29^

Finally, the findings of the present study support the notion that schizophrenia is a heritable disease and this has important implications. Firstly, it encourages the search for the genetic variants or molecular bases for the disorder. Such findings will ultimately inform psychiatric nosology. Secondly, it may generate new models for prevention and treatment by encouraging clinicians to invest in research on early detection and treatment, by cost-effective public mental health education and establishment of ‘early psychosis’ programmes, especially for family members at higher risk for mental illness.

### Limitations of the study

A major limitation of this study is the use of family history method; although it saved cost and time, lack of sensitivity for many psychiatric disorders is a major drawback. Direct interview of all relatives, whilst having its problems, such as selection bias, could have made more rigorous diagnosis possible, especially for the control group. However, in this study the magnitude of this problem may have been attenuated by the use of multiple informants in the case of the schizophrenia probands.

Secondly, although our sample size was larger than that of previous studies from our region, a larger proband and control group sample size would have been probably advantageous.
